# P-96. Comparative Outcomes of Dalbavancin vs. Standard Intravenous Antibiotics in Staphylococcus aureus Osteomyelitis: A Retrospective, Propensity-Matched Cohort Study Using Real-World Data

**DOI:** 10.1093/ofid/ofaf695.325

**Published:** 2026-01-11

**Authors:** Paddy Ssentongo, Silvana Ribeiro Papp, Siddartha Guru, Zinaida Perciuleac, Cory M Hale, Tonya Crook, Anas Atrash, Poonam Bai

**Affiliations:** Penn State Health Milton S. Hershey Medical Center, Hershey, PA; UPMC, Dover, PA; Penn State Health Milton S. Hershey Medical Center, Hershey, PA; Penn State Health Milton S. Hershey Medical Center, Hershey, PA; Penn State Health Milton S. Hershey Medical Center, Hershey, PA; Penn State Hershey College of Medicine, Hershey, PA; UPMC, Dover, PA; Penn State Health Milton S. Hershey Medical Center, Hershey, PA

## Abstract

**Background:**

Osteomyelitis due to *Staphylococcus aureus* often requires prolonged intravenous antibiotic therapy. Dalbavancin, a long-acting lipoglycopeptide, may simplify management. This study compares incident clinical outcomes among patients treated with dalbavancin versus standard intravenous antibiotics for osteomyelitis.

**Figure 1.**
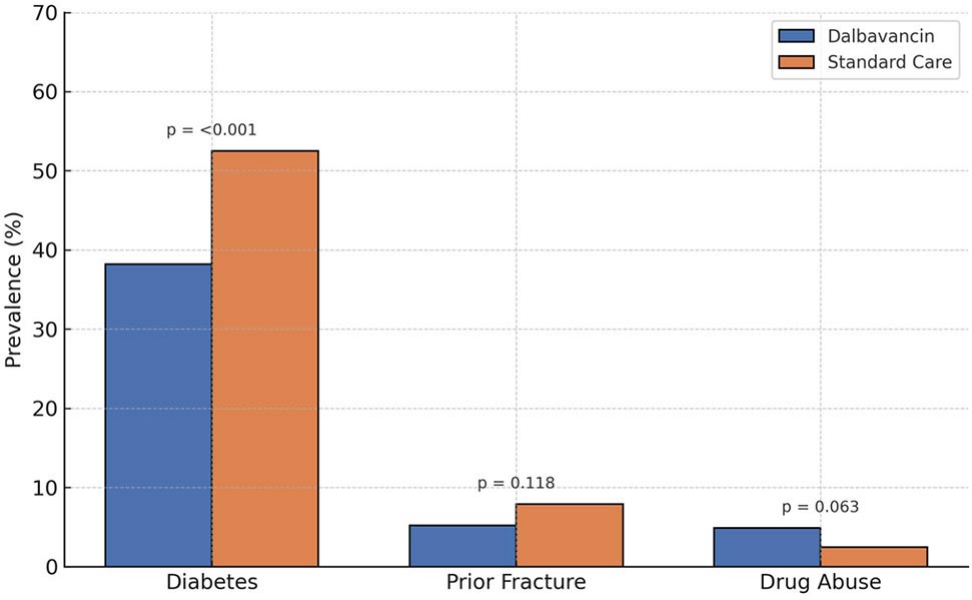
Baseline Comorbidity Burden by Treatment Group Prevalence of diabetes, prior fracture, and drug abuse among patients treated with dalbavancin versus standard intravenous therapy is shown. Bars represent group percentages with standard error bars.

**Figure 2:**
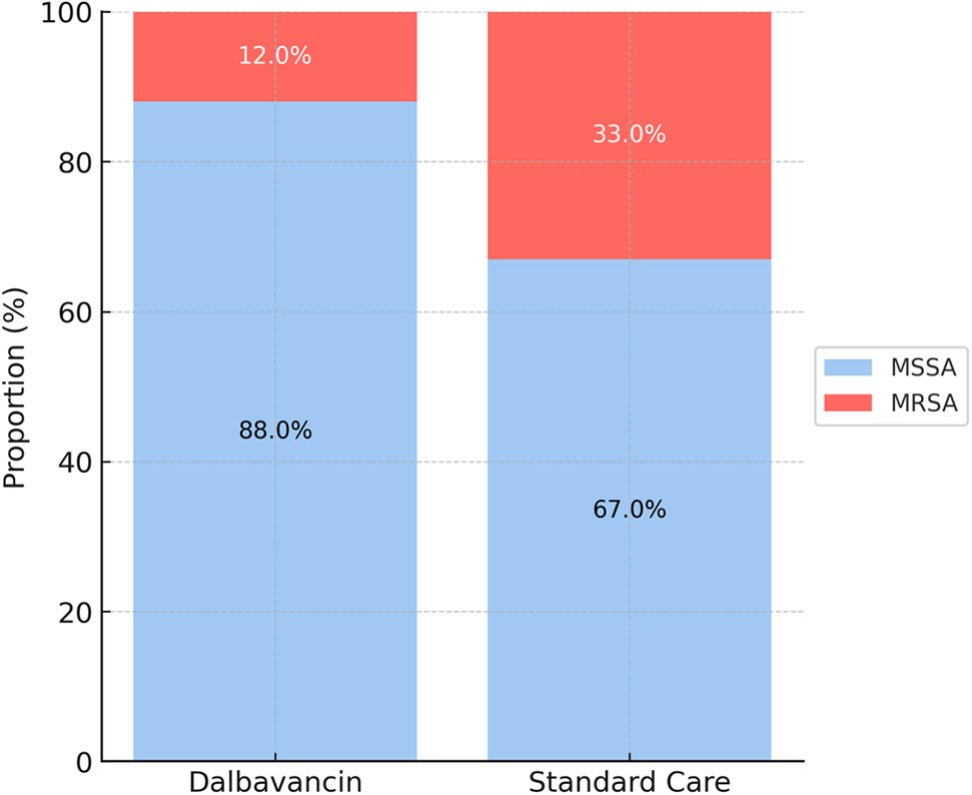
Distribution of MRSA and MSSA Infections by Treatment Group. Stacked bar chart displaying the proportion of methicillin-resistant (Staphylococcus aureus; MRSA) versus methicillin-susceptible (Staphylococcus aureus; MSSA) infections in patients treated with dalbavancin compared to those treated with standard intravenous antibiotics.

**Methods:**

We performed a retrospective analysis using the TriNetX Global Collaborative Network (141 healthcare organizations). Adults ≥18 years with a diagnosis of *S. aureus*-associated osteomyelitis and no bacteremia were included. Patients receiving dalbavancin (n = 406) were propensity-matched 1:1 to those treated with standard antibiotics (vancomycin, daptomycin, cefazolin, nafcillin, or linezolid). Outcomes were evaluated over 90 days following the index antibiotic exposure, limited to incident events (excluding patients with the outcome prior to the window). Kaplan–Meier survival, risk differences, and hazard ratios were calculated.

**Figure 3.**
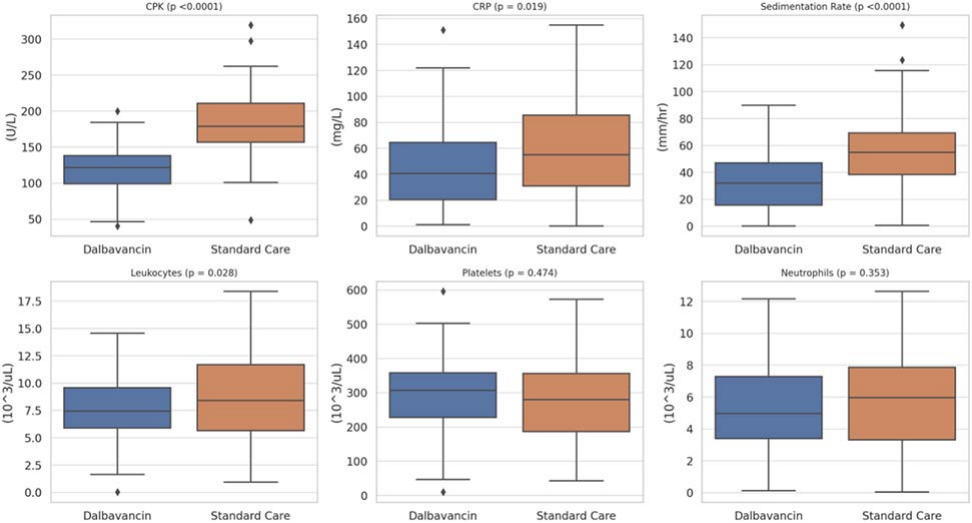
Distribution of Continuous Laboratory Outcomes by Treatment Group. Boxplots display the distribution of selected laboratory markers, including C-reactive protein, erythrocyte sedimentation rate, leukocyte count, platelet count, creatine phosphokinase, and neutrophil count, among patients treated with dalbavancin or standard intravenous antibiotics. Each panel includes p-values derived from unpaired t-tests comparing groups.

**Figure 4.**
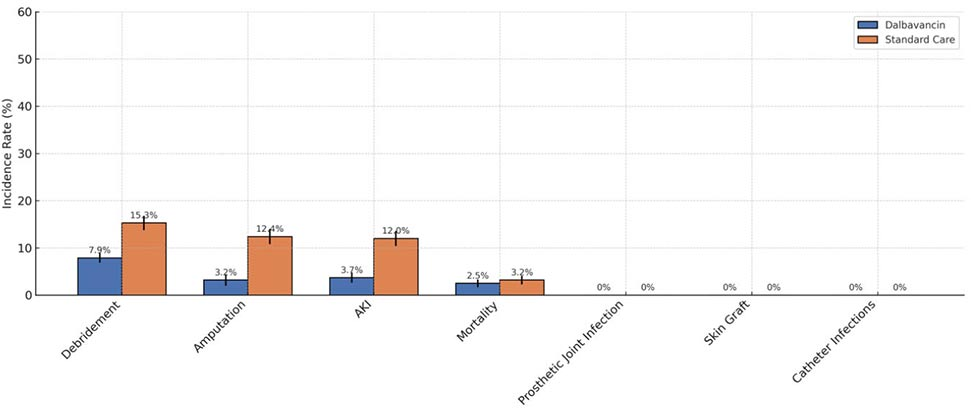
Incident Outcomes within 90 Days. This bar chart compares the 90-day incidence rates of key clinical outcomes between patients treated with dalbavancin and those treated with standard intravenous antibiotics for Staphylococcus aureus osteomyelitis. Outcomes are presented in descending order of frequency in the standard care group. Bars represent incidence rates with standard error bars, and outcomes with 0% incidence are labeled accordingly.

**Results:**

Matched cohorts were similar in age (mean 48.6 years), sex (70.4% men), and obesity prevalence (23.2%). Diabetes prevalence was lower in the dalbavancin group than standard of care (38.2% vs. 52.5%, Fig 1). MRSA accounted for 12.0% of cases in the dalbavancin group and 33.0% in the standard care group (Fig 2). Dalbavancin was associated with significantly lower risks of amputation (3.2% vs. 12.4%; HR, 0.15; 95% CI, 0.06–0.35; *P*< 0.001) and acute kidney injury (3.7% vs. 12.0%; HR, 0.23; 95% CI, 0.11–0.50; *P*< 0.001, Fig 3). Average C-reactive protein levels during treatment were lower in the dalbavancin group compared to standard care (19.6 mg/L vs. 43.1 mg/L, Fig 4). No differences were observed in catheter-related infections or skin graft complications. Ninety-day mortality was lower in the dalbavancin group (2.5% vs. 3.2%; HR, 0.31; 95% CI, 0.10–0.94; *P*=0.029).

**Conclusion:**

In this propensity-matched cohort study, dalbavancin was associated with lower rates of amputation, acute kidney injury, and other infectious complications in patients with *S. aureus* osteomyelitis. These findings support dalbavancin as a safe and effective alternative to standard intravenous therapy.

**Disclosures:**

All Authors: No reported disclosures

